# Treatment of Pancreatic Cancer Using Near-Infrared Photoimmunotherapy Targeting Cancer-Associated Fibroblasts in Combination with Anticancer Chemotherapeutic Drug

**DOI:** 10.3390/cancers17091584

**Published:** 2025-05-07

**Authors:** Hiroki Yonemura, Masaki Kuwatani, Kohei Nakajima, Atsushi Masamune, Mikako Ogawa, Naoya Sakamoto

**Affiliations:** 1 Department of Gastroenterology and Hepatology, Hokkaido University Faculty of Medicine and Graduate School of Medicine, North 15, West 7, Kita-ku, Sapporo 060-8648, Hokkaido, Japan; kyokui100055@gmail.com (H.Y.); sakamoto@med.hokudai.ac.jp (N.S.); 2 Laboratory of Bioanalysis and Molecular Imaging, Graduate School of Pharmaceutical Sciences, Hokkaido University, North 12, West 6, Kita-ku, Sapporo 060-0812, Hokkaido, Japan; knakajima@pharm.hokudai.ac.jp (K.N.); mogawa@pharm.hokudai.ac.jp (M.O.); 3 Division of Gastroenterology, Tohoku University Hospital, Seiryocho1-1, Aoba-ku, Sendai 980-8574, Miyagi, Japan; manager@gastroente.med.tohoku.ac.jp; 4 Institute for Chemical Reaction Design and Discovery (WPI-ICReDD), Hokkaido University, North 21, West 10, Kita-ku, Sapporo 001-0021, Hokkaido, Japan

**Keywords:** cancer-associated fibroblast, near-infrared photo-immunotherapy, pancreatic ductal cancer, combination therapy

## Abstract

**Background**: Pancreatic ductal adenocarcinoma (PDAC), which has a poor prognosis, involves an overabundance of fibroblasts and extracellular matrix. Cancer-associated fibroblasts (CAFs) are critical for providing structural support by secreting soluble factors and extracellular matrix proteins into the stroma. We assessed the potential of near-infrared photoimmunotherapy (NIR-PIT) targeting CAFs in PDAC. **Methods**: PDAC cells (Capan-1 and SUIT-2) and CAFs (hPSC-5) were used. Anti-human fibroblast activation protein (FAP)/podoplanin (PDPN) antibodies were used to bind to CAFs and conjugates with the specific photosensitizer IRDye^®^700DX (IR700) to investigate the effects of NIR-PIT. Thereafter, BALB/c Slc-*nu/nu* mice were transplanted with Capan-1 and/or CAFs and treated with gemcitabine (GEM) with or without NIR-PIT. **Results**: The binding rate of anti-FAP antibody-AlexaFluor^®^488 conjugate to hPSC-5 cells was high, whereas that of the anti-PDPN antibody-conjugate was not. The incubation of anti-FAP antibody-IR700 conjugate (αFAP-IR700) with hPSC-5 cells for 3 h led to maximal fluorescence on the surface of hPSC-5 cells. When NIR-PIT with αFAP-IR700 was performed in the co-culture group of Capan-1 and hPSC-5 cells, the proliferative capacity of Capan-1 cells decreased to the same level as that when Capan-1 cells were cultured alone (*p* < 0.05). In vivo, compared with the GEM group, the NIR-PIT with the GEM group showed a significant reduction in the tumor volume (day 28: 79 vs. 382 mm^3^, *p* < 0.05). Tumor volumes in the NIR-PIT group were not reduced compared with those in the control group. **Conclusions**: Combining NIR-PIT with conventional chemotherapy to target CAFs may enhance the anticancer effects on PDAC.

## Simple Summary

Pancreatic ductal adenocarcinoma (PDAC) has a poor prognosis due to its dense stroma rich in cancer-associated fibroblasts (CAFs), which promote tumor progression and treatment resistance. This study evaluated near-infrared photoimmunotherapy (NIR-PIT), targeting CAFs in PDAC. An anti-fibroblast activation protein (FAP) antibody conjugated to IRDye^®^700DX (αFAP-IR700) was tested on pancreatic cancer cells and hPSC-5 (CAFs). αFAP-IR700 successfully bound to hPSC-5, inducing cell death by NIR-PIT. Pancreatic cancer cells exhibited enhanced proliferation when co-cultured with hPSC-5, but this effect was reduced by NIR-PIT. In vivo, combining NIR-PIT with gemcitabine (GEM) significantly reduced tumor volume compared to GEM alone. These results suggest that targeting stromal cells with NIR-PIT enhances the efficacy of chemotherapy, providing a promising therapeutic strategy for PDAC.

## 1. Introduction

The 5-year survival rate of patients with pancreatic ductal adenocarcinoma (PDAC) remains low. In Japan, the survival rate is less than 10%, with similarly high morbidity and mortality [1,2,3]. The hallmark of PDAC is an extensive desmoplastic reaction characterized by dense stroma with an overabundance of cancer-associated fibroblasts (CAFs) and extracellular matrix. The dense stroma of PDAC creates a physical barrier to systemic chemotherapy, thus impairing effective delivery of chemotherapeutic drugs to cancer cells [4]. In PDAC, CAFs are derived from activated pancreatic stellate cells (PSCs) through a process triggered by signaling molecules such as interleukin-6, platelet-derived growth factor, and transforming growth factor-β [5]. These molecules are secreted by cancer cells and PSCs; thus, activation of PSCs to CAFs can be accelerated in tumors. PSCs/CAFs contribute to tumor progression by secreting various factors that enhance tumor cell proliferation, promote metastasis, and suppress anticancer immune responses [6]. Thus, PSCs and CAFs are potential therapeutic targets for patients with PDAC.

Many approaches targeting the stroma and/or CAFs in tumors have been reported. For example, hyaluronidase was used to reduce cancer stroma, resulting in decreases in collagen, proteoglycan, glycoprotein, and hyaluronan cross-linking in the stroma and enhanced chemotherapeutic effects [7,8]. Other studies using murine cancer models demonstrated cancer suppression effects by inhibiting the sonic hedgehog pathway, which activates the conversion of PSCs into CAFs [9]. This inhibition enhances the effectiveness of gemcitabine (GEM), a key chemotherapeutic drug for PDAC. Promising results were obtained in preclinical studies; however, phase Ib/II trials showed no additional therapeutic benefits [10]. Although other approaches targeting the stroma and CAFs in tumors have been investigated, none of these strategies have been established for the clinical treatment of PDAC, likely because of differences in the tumor microenvironment between mice and humans and/or insufficient drug delivery caused by the dense stromal barrier. Therefore, novel approaches, such as antibodies with smaller molecules and adequate target attainment, are needed to treat PDAC.

Near-infrared photoimmunotherapy (NIR-PIT) uses a specific monoclonal antibody against a cancer-cell surface antigen conjugated with a photosensitizer IRDye^®^700DX (IR700). Binding of the antibody-IR700 conjugate to target cells and followed by irradiation with near-infrared (NIR) light rapidly induces cell death via the formation of insoluble aggregates on the cell membrane. This aggregation physically damages the cell membrane, and an influx of water into the cell causes cell swelling and death [11,12]. NIR-PIT targeting EGFR-expressing cancer cells was approved for patients with inoperable head and neck cancer in Japan in 2021. In preclinical studies of esophageal, lung, and mammary cancer models, NIR-PIT targeting CAFs was effective in vitro and in vivo [13,14,15]. These studies targeted the anti-fibroblast activation protein (FAP), which is highly expressed on the CAF surface [8], and used an anti-FAP antibody conjugated with IR700. In addition, NIR-PIT targeting CAFs enhanced the therapeutic effects of 5-fluorouracil without adverse effects in an esophageal cancer model [16]. As PDAC harbors a richer stroma, the so-called abundant desmoplasia, compared with esophageal, lung, and mammary cancer, NIR-PIT targeting CAFs combined with anticancer drugs may yield more efficient results. However, preclinical studies are required for future clinical applications.

In this study, we first examined whether NIR-PIT could deplete CAFs/PSCs. We performed NIR-PIT using antibody-IR700 conjugates that target CAFs in PDAC. Because depletion of CAFs can reduce the stroma and enhance drug delivery to cancer cells, a combination therapy of NIR-PIT targeting CAFs and chemotherapy with GEM was secondly investigated in a murine PDAC model.

## 2. Materials and Methods

### 2.1. Cell Lines and Cell Culture

The PDAC cell lines, Capan-1 and SUIT-2, were purchased from the American Type Culture Collection (Manassas, VA, USA) and the Japanese collection of Research BioResources (Osaka, Japan), respectively. The hPSC-5 cells (RIKEN Bio Core Center, Kyoto, Japan) derived from human pancreatic cancer were used as PSCs/CAFs. hPSC-5 is pancreatic stellate cells extracted from human pancreatic cancer cells and is treated as CAFs in the previous literature [17,18]. PDAC cells were cultured in RPMI 1640 supplemented with 10% fetal bovine serum and 1% penicillin/streptomycin, and hPSC-5 cells were cultured at 37 °C in 5% CO_2_ using DMEM/Ham’s F-12 supplemented with 10% fetal bovine serum and 1% penicillin/streptomycin. The hPSC-5 cells are finite proliferating cells; therefore, they were used after 6–10 passages. Cell cultures were regularly tested for *Mycoplasma* infection using a polymerase chain reaction Mycoplasma Detection Set (Takara Bio, Shiga, Japan).

### 2.2. Antibodies and Reagents

Anti-human FAP antibody (clone 427819; R&D Systems, Minneapolis, MN, USA), anti-PDPN antibody (clone 101AP; Life Technologies, Carlsbad, CA, USA), IRDye^®^ 700DX (IR700; Li-COR Bioscience, Lincoln, NE, USA), anti-calreticulin antibody-Alexa Fluor^®^ 488 (ab196158; Abcam, Cambridge, UK), Alexa Fluor^®^ 488 (Life Technologies), and gemcitabine hydrochloride (GEM; Eli Lilly, Indianapolis, IN, USA) were used.

### 2.3. Animals

All experiments using mice were conducted in accordance with ‘Hokkaido University Regulations Concerning Animal Experimentation’. The animal experimental protocol was approved and reviewed by the Ethics Review Committee for Animal Experiments at Hokkaido University (15-0119). Six-week-old female nude mice (BALB/c Slc-*nu/nu*; Japan SLC, Inc., Shizuoka, Japan) were purchased and raised on alfalfa-free feed to reduce autofluorescence in animals during fluorescence imaging for 4–5 days before the experiments.

### 2.4. Preparation of Antibody-Dye Conjugates

Anti-human FAP or PDPN antibody and dyes were incubated at 20–25 °C for 3 h in 0.1 M Na_2_HPO_4_ (pH 8.5). The sample was purified using Amicon Ultra (30 K: Merck, Kenilworth, NJ, USA) and a centrifuge (Himac CR-N; Eppendorf Himac Technologies, Hamburg, Germany) at 4 °C and 2000 × *g*. Only anti-human PDPN antibody-IR700 was purified using Hitrap Protein G HP columns (Global Life Sciences Technologies Japan K.K., Tokyo, Japan). The concentration of the complex was determined using a bicinchoninic acid assay kit (Thermo Fisher Scientific, Waltham, MA, USA) by measuring the absorbance at 562 nm using a plate reader (Infinite^®^ 200 PRO; Tecan, Männedorf, Switzerland). The IR700 and Alexa Fluor^®^ 488 concentration was calculated using a spectrophotometer (UV-1800; Shimadzu Corporation, Kyoto, Japan) at wavelengths of 689 nm and 495 nm, respectively. Approximately 3–4 and 7 dye molecules were conjugated in the antibody-IR700 and antibody-Alexa Fluor^®^ 488 complexes, respectively.

### 2.5. FAP Expression in hPSC-5

The hPSC-5 cells were seeded at a density of 3.0 × 10^5^ cells/dish in a 35 mm dish and incubated for 24 h. The cells were washed with phosphate-buffered saline (PBS) and the medium was replaced, and then anti-human FAP antibody-Alexa Fluor^®^ 488 complex was added to a concentration of 5 µg/mL and incubated for 3 h at 37 °C. The cells were collected using trypsin, and fluorescence intensity was measured using flow cytometry (Gallios; Beckman Coulter, Brea, CA, USA). Data obtained by flow cytometry were analyzed using Kaluza Analysis 2.1 software (Beckman Coulter).

### 2.6. In Vitro NIR-PIT for hPSC-5

#### 2.6.1. Qualitative Evaluation Using a Fluorescence Microscope

The hPSC-5 cells were seeded into a 35 mm dish at a density of 3.0 × 10^5^ cells/dish and incubated for 24 h. The cells were washed with PBS, the medium was replaced, and anti-human FAP antibody-IR700 conjugate (αFAP-IR700) was added to a concentration of 5 µg/mL. The cells were incubated for 1, 3, 6, 12, and 24 h at 37 °C. After washing with PBS, the cells were observed under a fluorescence microscope (CKX41; Olympus, Tokyo, Japan). For fluorescence observation, the excitation filter was set to 673–748 nm for IR700, and the fluorescence filter was set to 765–855 nm.

Ethidium homodimer-1 (EthD-1; Thermo Fisher Scientific) was added to a concentration of 1 µg/mL. The cells were then irradiated with NIR light through the excitation filter of a microscope (673–748 nm) for 120 s (18 J/cm^2^). At 3 h after irradiation, the cells were observed using an excitation filter at 480–550 nm and a fluorescence filter at 590 nm to determine the number of dead cells. Images obtained using the fluorescence microscope were analyzed using ImageJ 1.53 software (accessed on 22 October 2022 at http://rsb.info.nih.gov/ij/).

#### 2.6.2. Quantitative Analysis of Dead Cells in NIR-PIT by Flow Cytometry

The hPSC-5 cells were seeded at a density of 3.0 × 10^5^ cells/dish in a 35 mm dish and incubated for 24 h. The cells were washed with PBS, and the medium was replaced. αFAP-IR700 was added to the dish at a concentration of 5 µg/mL, followed by incubation for 3 h. NIR light from a light-emitting diode (LED) [670–710 nm (L690D-66-16100, Epitex, Inc., Singapore)] was irradiated at 5, 25, and 50 J/cm^2^. The power density was determined using an optical power meter (PM100, Thorlabs, Newton, NJ, USA; 50 mW/cm^2^). After 3 h, the cells were collected, suspended in PBS, and incubated with 0.01 mg/mL propidium iodide (Life Technologies) at 20–25 °C for 15 min. Dead cells were detected using flow cytometry, and the data were analyzed as described above.

### 2.7. Co-Culture of hPSC-5 and PDAC Cells

The cells were divided into three groups: PDAC alone, co-cultured PDAC and hPSC-5 cells, and co-cultured PDAC and hPSC-5 cells with NIR-PIT using αFAP-IR700. The cells were seeded into 35 mm dishes (PDAC: 1.0 × 10^5^ cells/dish and hPSC-5: 1.0 × 10^5^ cells/dish). The anti-human FAP antibody-IR700 conjugate was added to the dish at a concentration of 5 µg/mL and incubated for 3 h. NIR light from an LED was irradiated at 50 J/cm^2^ (670–710 nm, 50 mW/cm^2^) on day 1. On days 1 (just before NIR-PIT), 3, and 5, the number of PDAC cells in five random fields of view at low magnification (20×) was counted under a fluorescent microscope (CKX41; Olympus), and the average of four dishes was calculated. Images obtained using the fluorescence microscope were analyzed as described above.

### 2.8. Confirmation of the Presence of Immunogenic Cell Death in NIR-PIT for hPSC-5 In Vitro

The hPSC-5 cells were seeded at a density of 3.0 × 10^5^ cells/dish in a 35 mm dish and incubated for 24 h. After washing with PBS and changing the medium, the dish was incubated with 5 µg/mL αFAP-IR700 for 3 h. NIR light from an LED (670–710 nm, 50 mW/cm^2^) was used for irradiation at 5, 25, and 50 J/cm^2^.

The following three assays were performed in the same way up to this step.

#### 2.8.1. Adenosine Triphosphate Assay

The supernatant was collected 3 h after the end of NIR irradiation and centrifuged at 4 °C at 5000× *g* for 5 min. The adenosine triphosphate (ATP) concentration in the supernatant after centrifugation was measured using an ATP assay kit (ENLITEN; Promega, Madison, WI, USA).

#### 2.8.2. Calreticulin Assay

At 3 h after NIR irradiation, the cells were collected, suspended in PBS, and incubated with anti-calreticulin antibody-Alexa Fluor^®^ 488 at 20–25 °C for 45 min. Fluorescence intensity was measured using flow cytometry.

#### 2.8.3. High Mobility Group Box 1 Assay

At 24 h after the end of NIR irradiation, the supernatant was collected and centrifuged at 4 °C at 300× *g* for 3 min. After centrifugation, the supernatant was concentrated 10-fold using an Amicon Ultra (10 K) and centrifuged, and the concentration of high-mobility group box 1 (HMGB1) was measured using a Human HMGB1 enzyme-linked immunosorbent assay kit (ARG81185, Arigo, Zhubei City, China).

### 2.9. In Vivo NIR-PIT for PDAC with CAF Using Capan-1 and hPSC-5 Cells in Murine Models

One previous study revealed that the tumor in the BALB/c nude mouse model inoculated with Capan-1 harbored a similar morphological structure to the primary human PDAC [19]. Therefore, a mixture of Capan-1 and hPSC-5 cells or Capan-1 cells alone was transplanted into the right buttocks of 6-week-old female BALB/c Slc-*nu/nu* mice at a density of 3.0 × 10^6^ cells per cell line. When the average tumor volume reached around 80 mm^3^, the mice were divided into the five groups: no treatment (NT) group as control, Capan-1 group as another control with Capan-1 cells alone and no treatment, GEM group as GEM monotherapy, NIR-PIT group as NIR-PIT monotherapy using αFAP-IR700, and NIR-PIT with GEM group as a combination therapy with GEM and PIT using αFAP-IR700. GEM (1 mg/mouse) and αFAP-IR700 (100 µg/mouse) were administered via the tail vein on days 1, 8, and 15. In the PIT group, the tumors were irradiated with NIR laser light (MLL-III-690–800 mW; Changchun New Industries Optoelectronics Tech Co., Jilin, China) at 75 J/cm^2^/mouse (150 mW/cm^2^, 500 s) 24 and 48 h after intravenous administration. Fluorescence images were obtained using an IVIS imaging system (PerkinElmer, Waltham, MA, USA) before and after light irradiation. The tumor volume was calculated using the formula (long diameter × short diameter^2^)/2, and changes were evaluated.

### 2.10. Statistical Analysis

Data are represented as the mean ± standard error of the mean (*n* > 3). All statistical analyses were performed using EZR version 1.68 (Saitama Medical Center, Jichi Medical University) [20], which is a graphical user interface for R (R Foundation for Statistical Computing). A one-way analysis of variance and Dunnett’s test were used for multiple comparisons. *p* values < 0.05 were considered to indicate significant results.

## 3. Results

### 3.1. FAP Expression on hPSC-5 Cells

**Figure 1:**
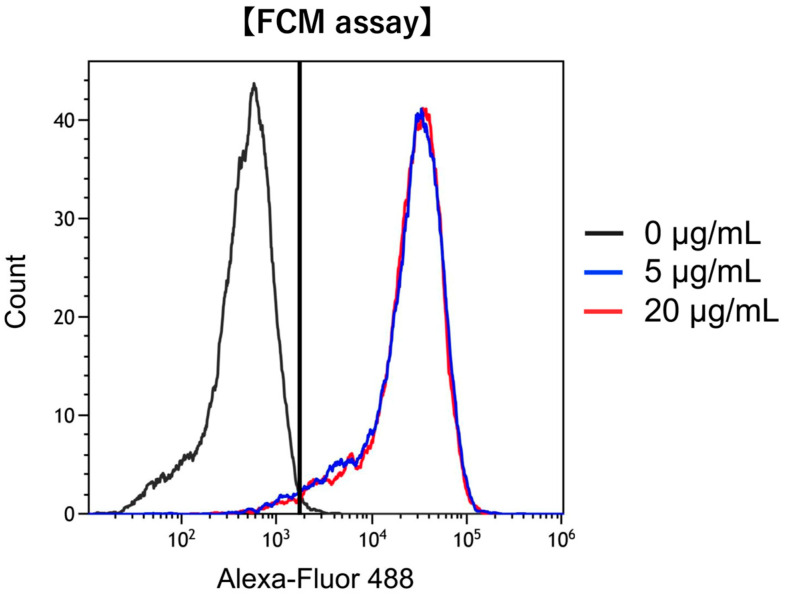
Confirmation of FAP expression in hPSC-5. Binding of anti-human FAP-Alexa Fluor^®^488 was observed in hPSC-5 cells, confirming that FAP antigens are expressed on the surface of hPSC-5 cells. black: control, blue: 5 µg/mL, red: 20 µg/mL.

To confirm whether FAP was expressed on hPSC-5 cells as observed on CAFs, we used anti-FAP antibody-AlexaFluor^®^488 conjugate and performed quantitative analysis using flow cytometry. As shown in Figure 1, the binding rate of anti-FAP antibody-AlexaFluor^®^488 conjugate at 5 µg/mL to hPSC-5 cells was 94%, whereas that at 20 µg/mL was 95%. The binding rate did not change even when the reagent concentration was changed to 5 µg/mL and 20 µg/mL, indicating that FAP was sufficiently expressed on the hPSC-5 cell surface.

### 3.2. Binding of Anti-FAP/PDPN Antibody-IR700 to hPSC-5 Cells

**Figure 2:**
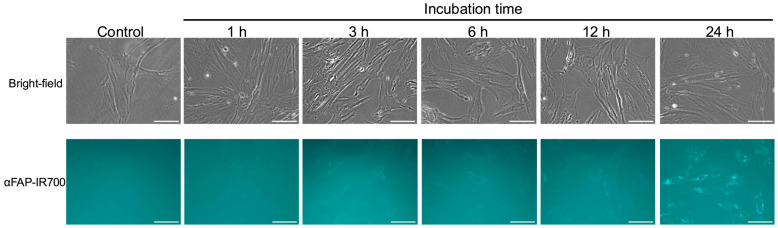
Confirmation of binding αFAP-IR700. Fluorescence microscopy was performed at 1, 3, 6, 12, and 24 h after co-culture of αFAP-IR700 with hPSC-5 cells. Binding of αFAP-IR700 to hPSC-5 cells was confirmed at all co-culture times. Scale bar: 100 µm (original magnification, 20×).

After confirmation of sufficient FAP expression on the hPSC-5 cell surface by the anti-FAP antibody-AlexaFluor^®^488 conjugate, we also investigated whether αFAP-IR700 and the anti-PDPN antibody-IR700 conjugate were bound to hPSC-5 using a fluorescence microscope. As the incubation time of αFAP-IR700 with hPSC-5 cells increased, fluorescence intensity also increased (Figure 2). Although fluorescence was detected after 1 h of incubation, maximal fluorescence was observed on the surface of hPSC-5 cells after 3 h of incubation. After 6 h of incubation, bright granular spots were observed. These results indicate that αFAP-IR700 was internalized into lysosomes over time. Namely, binding of αFAP-IR700 and the anti-FAP antibody-AlexaFluor^®^488 conjugate to hPSC-5 cells was also observed (Figure 2). Fluorescence due to bindings of αFAP-IR700 and anti-PDPN antibody-IR700 conjugate to PDAC cells was not detected (Figure S1). Unfortunately, fluorescence due to binding of the anti-PDPN antibody-IR700 conjugate to hPSC-5 cells was also not observed (Figure S2).

### 3.3. In Vitro NIR-PIT for hPSC-5 Cells

#### 3.3.1. Fluorescence Microscopy

**Figure 3:**
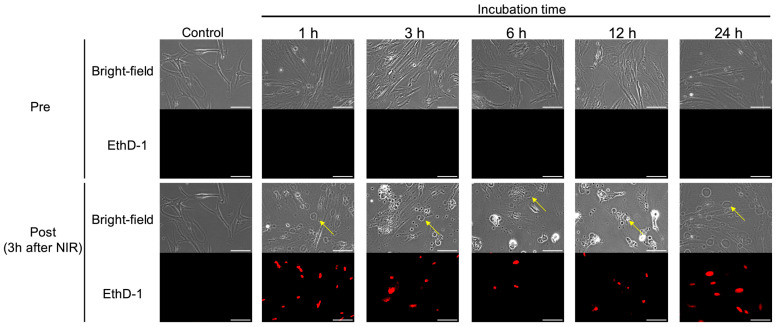
Microscopic observation of NIR-PIT for hPSC-5 cells using αFAP-IR700 in vitro. At 3 h after the end of irradiation, cell swelling (yellow arrow) and nuclear staining with EthD-1 (red) were observed under a bright-field and fluorescence microscope, indicating cell death induced by NIR-PIT at all incubation times. Scale bar: 100 µm (original magnification, 20×).

The effects of NIR-PIT on hPSC-5 cells in vitro were confirmed. As shown in Figure 3, swelling of hPSC-5 cells after NIR-PIT was observed using bright-field microscopy at all incubation times. After NIR irradiation, cell swelling began immediately (Figure S3a,b), and cell death was also observed at all incubation times after 1 h of nuclear staining with EthD-1 (Figure 3). At 3 h after NIR irradiation, cell death was observed in all cells in the irradiated field of view. In the non-irradiated field, neither cell swelling nor nuclear staining by EthD-1 was observed even 3 h after NIR irradiation, indicating that cell death was induced only in the irradiated field (Figure S3c–e).

#### 3.3.2. Flow Cytometry

**Figure 4:**
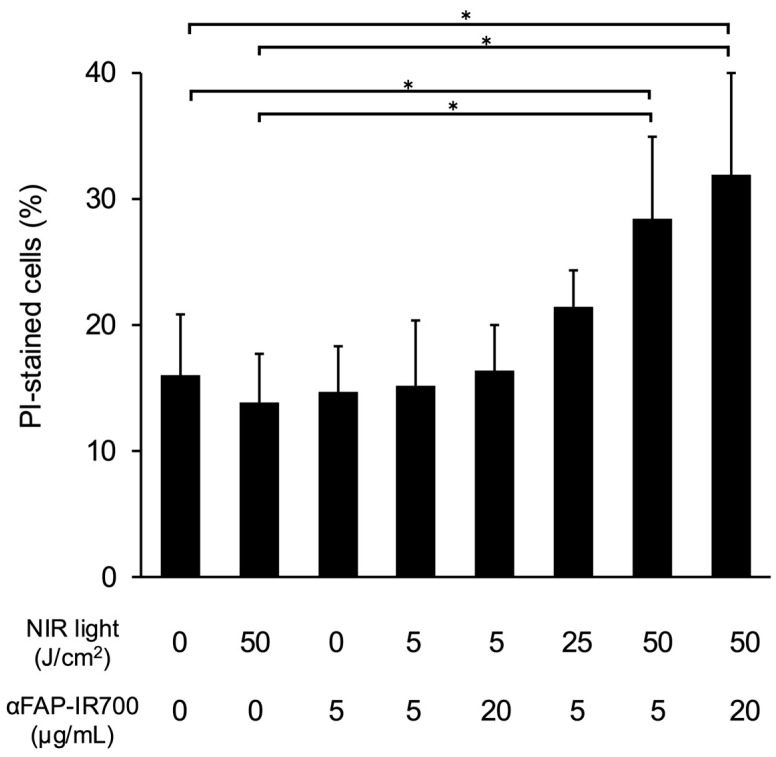
Flow cytometry of NIR-PIT for hPSC-5 cells using αFAP-IR700 in vitro. NIR-PIT was performed on hPSC-5 cells using αFAP-IR700. The cells were collected 3 h after irradiation, and the percentage of dead cells was calculated as the mean value ± standard error of the mean using propidium iodide (PI) staining. Compared with the control group, the percentage of dead cells was significantly higher in the NIR-PIT group at 50 J/cm^2^ (*n* = 5, * *p* < 0.05 vs. untreated control). No differences were observed between the reagent concentrations.

Figure 4 shows the quantitative evaluation of the effect of NIR-PIT on hPSC-5 cells using αFAP-IR700 by flow cytometry. Compared with the control group (αFAP-IR700, 0 μg/mL with NIR, 0 J/cm^2^), significant cell death was observed in the NIR-PIT group (αFAP-IR700, 5 and 20 µg/mL with NIR, 50 J/cm^2^) (*n* = 5, * *p* < 0.05 vs. control). In addition, cell death was observed in the NIR-PIT group with lower irradiation (αFAP-IR700, 5 μg/mL with NIR, 25 J/cm^2^), although the level of cell death was not significant compared with that in the control (*n* = 5, *p* = 0.31 vs. control). There were no significant differences between the other groups and the control (NIR, 50 J/cm^2^ alone, *p* = 1; αFAP-IR700, 5 μg/mL alone, *p* = 1; αFAP-IR700, 5 μg/mL with NIR, 5 J/cm^2^, *p* = 1; αFAP-IR700, 20 μg/mL with NIR, 5 J/cm^2^, *p* = 1).

### 3.4. Effect of NIR-PIT on PDAC Cell Proliferation In Vitro

**Figure 5:**
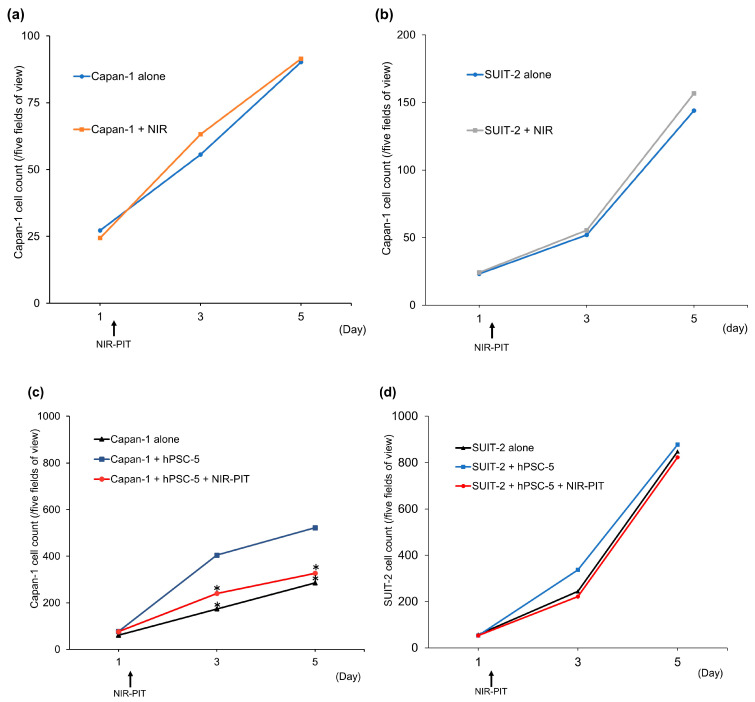
Confirmation of PDAC cell proliferative ability. (**a**) Capan-1 cells without hPSC cells similarly proliferated irrespective of NIR-PIT using αFAP-IR700. (**b**) SUIT-2 cells without hPSC cells also similarly proliferated irrespective of NIR-PIT using αFAP-IR700. (**c**) Capan-1 cells co-cultured with hPSC-5 cells proliferated faster than Capan-1 cells cultured alone. When hPSC-5 cells were treated with NIR-PIT, the proliferative ability of Capan-1 cells decreased to the same level as that of the Capan-1 cell group (each group, *n* = 4, * *p* < 0.05 vs. Capan-1 cells alone). (**d**) In SUIT-2, there was no effect of hPSC-5 and NIR-PIT on cell proliferation of SUIT-2 (each group, *n* = 4, *p* = 1 vs. SUIT-2 cells alone).

To investigate whether the interaction between hPSC-5 and PDAC cells increases the proliferative potential of PDAC cells, and whether inhibition of hPSC-5 can reduce the proliferative potential of PDAC cells, NIR-PIT was performed on Capan-1, SUIT-2, and hPSC-5 cells using αFAP-IR700 co-cultured with Capan-1 and SUIT-2 cells in vitro (Figure 5). Both Capan-1 and SUIT-2 cells without hPSC-5 similarly increased irrespective of NIR-PIT using αFAP-IR700 (Figure 5a,b). Compared with Capan-1 cells alone, co-culture of Capan-1 and hPSC-5 cells led to a significant increase in the number of Capan-1 cells (*n* = 4, * *p* < 0.05) (Figure 5c). When NIR-PIT with αFAP-IR700 was performed in the co-culture group, the proliferative capacity of Capan-1 cells decreased to the level of that of Capan-1 cells alone (*n* = 4, * *p* < 0.05) (Figure 5c). In SUIT-2, there were no differences in cell proliferative capacity among all groups (Figure 5d).

### 3.5. Immunogenic Cell Death in NIR-PIT for hPSC-5 Cells

**Figure 6:**
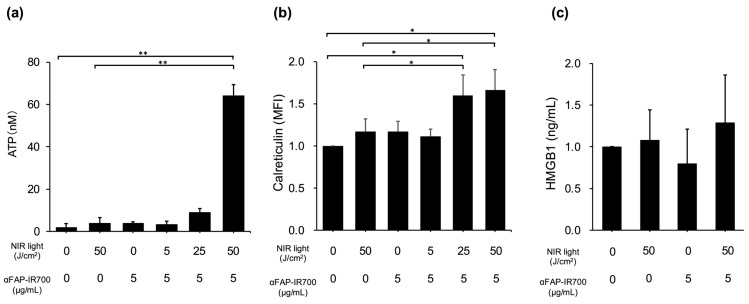
Confirmation of ICD in hPSC-5 using αFAP-IR700 in NIR-PIT. (**a**) In the ATP assay, the NIR-PIT group with 50 J/cm^2^ and αFAP-IR700 (5 μg/mL) showed a significant increase in extracellular ATP release (*n* = 3, ** *p* < 0.01 vs. control). (**b**) In the calreticulin assay, the NIR-PIT groups with 25 and 50 J/cm^2^ and αFAP-IR700 (5 μg/mL) showed a significant increase in calreticulin expression (*n* = 3, * *p* < 0.05 vs. control). (**c**): In the HMGB1 assay, there was a slight tendency for HMGB1 to be released in the NIR-PIT group at 50 J/cm^2^ (*n* = 3, *p* = 0.70 vs. the untreated control).

To confirm immunogenic cell death (ICD) by NIR-PIT for hPSC-5 cells, calreticulin translocation and release of ATP/HMGB1 were quantified in hPSC-5 cells after NIR-PIT. Compared with the control group (αFAP-IR700, 0 μg/mL with NIR, 0 J/cm^2^), the NIR-PIT group with 50 J/cm^2^ and αFAP-IR700 (5 μg/mL) showed significant release of ATP into the extracellular space (*n* = 3, ** *p* < 0.01). There were no significant differences between the other treatment groups and control group (NIR, 50 J/cm^2^ alone, *p* = 1; αFAP-IR700, 5 μg/mL alone, *p* = 1; αFAP-IR700, 5 μg/mL with NIR, 5 J/cm^2^, *p* = 1; αFAP-IR700, 5 μg/mL with NIR, 25 J/cm^2^, *p* = 1) (Figure 6a). The NIR-PIT groups with 25 and 50 J/cm^2^ irradiation showed a significant increase in calreticulin expression on the cell membrane (*n* = 3, * *p* < 0.05 vs. control). In the NIR-PIT group with 5 J/cm^2^, there was a trend towards calreticulin membrane surface migration, but the difference was not significant (*n* = 3, *p* = 0.06 vs. control). There were no significant differences between the other treatment groups and control (NIR, 50 J/cm^2^ alone, *p* = 1; αFAP-IR700, 5 μg/mL alone, *p* = 1) (Figure 6b). There was a slight but not significant tendency towards HMGB1 release in the NIR-PIT group at 50 J/cm^2^ (*n* = 3, *p* = 0.70 vs. control). No significant HMGB1 release was observed in the other treatment groups (Figure 6c).

### 3.6. In Vivo NIR-PIT for hPSC-5 Cells

**Figure 7:**
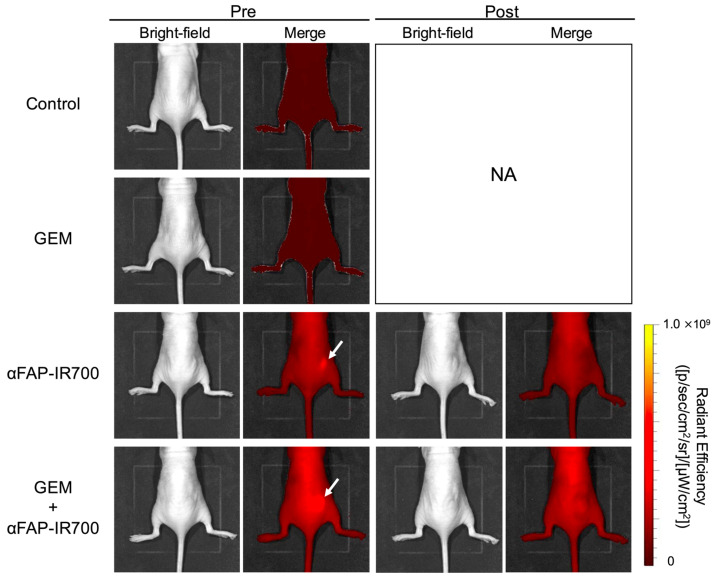
In vivo NIR-PIT by αFAP-IR700 in a murine model with Capan-1 + hPSC-5 cells. In both the αFAP-IR700 group and the NIR-PIT with GEM group, accumulation of αFAP-IR700 in the tumor was observed before NIR-PIT (white arrow). After NIR irradiation, fluorescence due to αFAP-IR700 accumulation disappeared in both groups. NA, not assessed.

The effect of NIR-PIT for CAFs with αFAP-IR700 on PDAC was investigated in murine models with Capan-1 + hPSC-5 cells. In both the NIR-PIT and NIR-PIT with GEM groups, accumulation of αFAP-IR700 in the tumor mass was observed 24 h after αFAP-IR700 injection. Immediately after NIR irradiation, the fluorescence from αFAP-IR700 disappeared in both groups, indicating that IR700 responded and aggregated after NIR irradiation (Figure 7).

**Figure 8:**
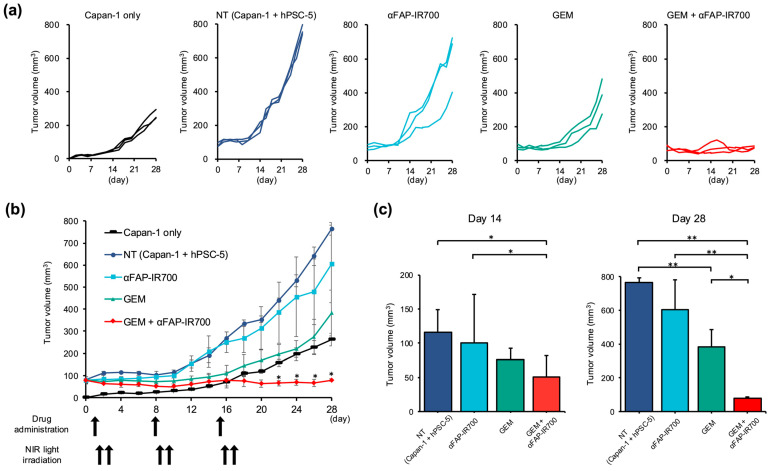
Tumor volumes of in vivo NIR-PIT by αFAP-IR700 in a murine model with Capan-1 + hPSC-5 cells. (**a**) Spider plot of Capan-1 cells alone, NT (Capan-1 + hPSC-5 cells), NIR-PIT with αFAP-IR700 monotherapy group, GEM alone, and combination groups with GEM plus NIR-PIT with αFAP-IR700, respectively. (**b**) Changes in tumor volume over time. Compared with the Capan-1 cell group, the NT group (Capan-1 + hPSC-5 cell group) showed significantly greater tumor growth. In addition, when the treatment was examined by type, the group that received NIR-PIT in combination with GEM showed a significant cancer-reducing effect compared to the group that received GEM alone (*n* = 3, * *p* < 0.05). (**c**) Comparison of tumor volume on treatment days 14 and 28: On day 14, tumor volume was significantly reduced in the GEM plus NIR-PIT group compared with that in the NT group and αFAP-IR700 monotherapy group. On day 28, tumor volume was significantly reduced in the GEM plus NIR-PIT group compared with that in the GEM group (*n* = 3, * *p* < 0.05, ** *p* < 0.01).

Spider plots of the tumor volumes for each group are shown in Figure 8a. The tumor of the NT group (transplanted with Capan-1 + hPSC-5 cells, *n* = 3) grew to 80 mm^3^ at the start of treatment, at 4 days after transplant (day 0 in Figure 8b), whereas the tumor mass of the Capan-1 group had not emerged at that time, and the mass emerged 6 days after transplant (15 mm^3^ on average) (day 2 in Figure 8b). Thereafter, the mass of the NT group continued to increase at a faster rate than in the Capan-1 alone group (day 28: 766 vs. 263 mm^3^, ** *p* < 0.01). On day 14, there was a significant reduction in tumor volume in the GEM with NIR-PIT group compared to that in the NT group (50.2 vs. 115 mm^3^; *n* = 3, * *p* < 0.05). The GEM group was not significantly different from the NT group at day 14 (75.9 vs. 115 mm^3^; *n* = 3, *p* = 0.09) (Figure 8c).

Compared with the GEM group, the NIR-PIT + GEM group showed a significant reduction in tumor size (day 28: 79 vs. 382 mm^3^; *n* = 3, * *p* < 0.05). The NIR-PIT monotherapy group showed no tumor reduction effects on the volume compared with the NT group (day 28: 605 vs. 766 mm^3^; *n* = 3, *p* = 0.78) (Figure 8c).

## 4. Discussion

This study demonstrated that NIR-PIT targeting CAFs, the primary source of stromal structures in PDAC, can enhance the efficacy of chemotherapy with GEM in vivo.

Although numerous studies reported that systemic administration of agents targeting stromal structures and/or CAFs reduced stroma within tumors [7,9,10], these agents may induce adverse effects in healthy tissues because of the lack of high specificity for stroma and/or CAFs. In contrast, the greatest advantage of NIR-PIT is its high selectivity for targeted cells, as it only kills cells that are bound to antibody-IR700 conjugates and irradiated with NIR light [21]. In the present study, significant cell death was induced in hPSC-5 cells as CAFs when they were irradiated with NIR light at 50 J/cm^2^. Because the expression level of FAP on CAFs is not extremely high, a relatively large light dose was required to induce NIR-PIT targeting CAFs compared to NIR-PIT targeting cancer cells [22]. A previous study showed that CAFs do not undergo cell death unless irradiated with light at doses of 25–50 J/cm^2^ [15], which is consistent with the present data. Cell death was observed only in areas irradiated with NIR light, indicating the selectivity of NIR-PIT. As the pancreas is a retroperitoneal organ located close to the stomach, duodenum, and large vessels, damage to other organs should be avoided. As NIR light irradiation does not show cytotoxicity in cells without IR700, NIR-PIT is a safe method for treating PDAC when appropriate light irradiation is performed. To irradiate tumors deep in the body with NIR light, we developed specialized irradiation devices, such as endoscopic nasobiliary/nasopancreatic drainage tubes [22]. This device can be applied to PDAC as well as biliary tract cancer; thus, NIR-PIT targeting CAFs in PDAC may be useful in patients with PDAC.

In PDAC cell-bearing mice, no significant tumor-suppressive effect was observed in mice treated with NIR-PIT targeting CAFs alone, whereas synergistic effects were induced by a combination of NIR-PIT targeting CAFs and GEM in PDAC. Thus, our results suggest that CAF reduction by NIR-PIT is an efficient method for enhancing the efficacy of anticancer chemotherapeutic drugs, including GEM, in PDAC. Although the current mainstream chemotherapies for PDAC are fluorouracil, leucovorin, irinotecan, and oxaliplatin (FOLFILINOX), as well as GEM with nab-paclitaxel, they are not tolerated by elderly or poorly conditioned patients because of their various adverse effects [23,24]. In our PDAC model, NIR-PIT targeting CAFs can reduce the cancer stroma, as well as in esophageal, lung, and mammary cancer models [14,15], leading to impairment of the stromal structure. Thus, NIR-PIT targeting of CAFs may improve the delivery of GEM to cancer cells. In addition, morphological changes such as cellular swelling were observed on the cell membrane immediately after NIR light irradiation, and cell death was completed 1 h after irradiation. The rapid cytotoxic effects of NIR-PIT may result in a therapeutic effect at an earlier phase than other treatments, such as chimeric antigen receptor T cells therapy targeting CAFs [25]. As PDAC progresses rapidly, NIR-PIT targeting CAFs is desired for PDAC.

Recently, activated CAFs were divided into three main subtypes, inflammatory CAFs, myofibrotic CAFs, and antigen-presenting CAFs, each of which has a variety of functions, including cancer-promoting and cancer-suppressing effects [8]. However, subtype-specific markers have not been identified, and the current grouping is based on the amount of alpha-smooth muscle actin expression [26,27]. In our study, although in vitro co-culture studies demonstrated that NIR-PIT with αFAP-IR700 significantly suppresses the proliferation of Capan-1 cancer cells, in vivo tumor suppression was not observed in the NIR-PIT with αFAP-IR700 alone group. The complexity of CAF subtypes in tumors may explain the differences between the in vitro and in vivo results. If surface markers specific to cancer-promoting CAFs are identified and monoclonal antibodies are established, NIR-PIT targeting the cancer-promoting CAFs would induce more significant therapeutic effects than the present NIR-P’IT with αFAP-IR700 in PDAC.

NIR-PIT targeted cancer induces ICD in cancer cells and subsequently activates immune responses. Therapeutic effects can occur in tumors in non-irradiated sites by anticancer immune cells, such as CD8^+^ T cells [28]. ICD is characterized by calreticulin translocation to the cell membrane and the release of damage-associated molecular patterns such as ATP and HMGB1 [29]. A previous report indicated that the ICD of inflammatory CAFs can also be caused by drugs and promoted by the stimulation of interferon-γ produced by CD8^+^ T cells and natural killer cells [30]. Furthermore, interferon-γ also activates antitumor immune responses, and thus, we investigated whether NIR-PIT with αFAP-IR700 induces ICD of CAFs. The extracellular release of ATP and calreticulin translocation were enhanced by NIR-PIT, but not the release of HMGB1. One possible reason for this observation is the instability of HMGB1 outside cells. The function and stability of HMGB1 change depending on its redox state; when it is completely oxidized, it is easily degraded [31]. Because antibody-IR700 generates reactive oxygen species and contributes to cell death [32,33], reactive oxygen species from IR700 may induce oxidation and degradation of HMGB1. However, considering previous data on ICD of cancer cells, we assume that the ICD of CAFs is also induced by NIR-PIT with αFAP-IR700. Thus, depletion of CAFs by NIR-PIT may exhibit therapeutic effects not only by reducing the stroma in PDAC but also by activating antitumor immunity. Further investigation of the allogeneic murine model would improve the understanding of the ICD of CAFs and subsequent antitumor immune responses.

## 5. Conclusions

In conclusion, we demonstrated the potential of combining NIR-PIT with conventional chemotherapy to enhance anticancer effects on PDAC. In future research, we will further optimize NIR-PIT and link it to clinical applications by maximizing treatment efficacy while minimizing adverse effects.

## Data Availability

The data presented in this study are available on request from the corresponding author. The data are not publicly available due to privacy laws.

## Supplementary Materials

The following supporting information can be downloaded at https://www.mdpi.com/article/10.3390/cancers17091584/s1; Figure S1: Fluorescence microscopy for confirmation of binding of anti-FAP (PDPN) antibody-IR700 conjugate to PDAC cells; Figure S2: Fluorescence microscopy for confirmation of binding of anti-PDPN antibody-IR700 conjugate to hPSC-5; Figure S3: Microscopic observation after NIR-PIT in vitro; Figure S4: Microscopic observation of NIR-PIT for human pancreatic cancer cells.

## Abbreviations

The following abbreviations are used in this manuscript:

αFAP-IR700	anti-human FAP antibody—IR700
ATP	adenosine triphosphate
CAFs	cancer associated fibroblasts
EthD-1	ethidium Homodimer—1
FAP	fibroblast activation protein
GEM	gemcitabine
HMGB1	high mobility group box 1
ICD	immunogenic cell death
IR700	IRDye^®^ 700DX
LED	light emitting diode
NIR	near-infrared
NIR-PIT	near-infrared photoimmunotherapy
NT	no treatment
PBS	phosphate buffered saline
PDAC	pancreatic ductal adenocarcinoma
PDPN	podoplanin
PSCs	pancreatic stellate cells
