# Supplementary material for: Treatment of Pancreatic Cancer Using Near-Infrared Photoimmunotherapy Targeting Cancer-Associated Fibroblasts in Combination with Anticancer Chemotherapeutic Drug
Source: Cancers (Basel). 2025 May 7;17(9):1584. doi: 10.3390/cancers17091584 (PMC12071749; doi:10.3390/cancers17091584)
Supplement: Supplementary file 1 [file cancers-17-01584-s001.zip › cancers-3575805-supplementary.pdf]

Supplementary Information

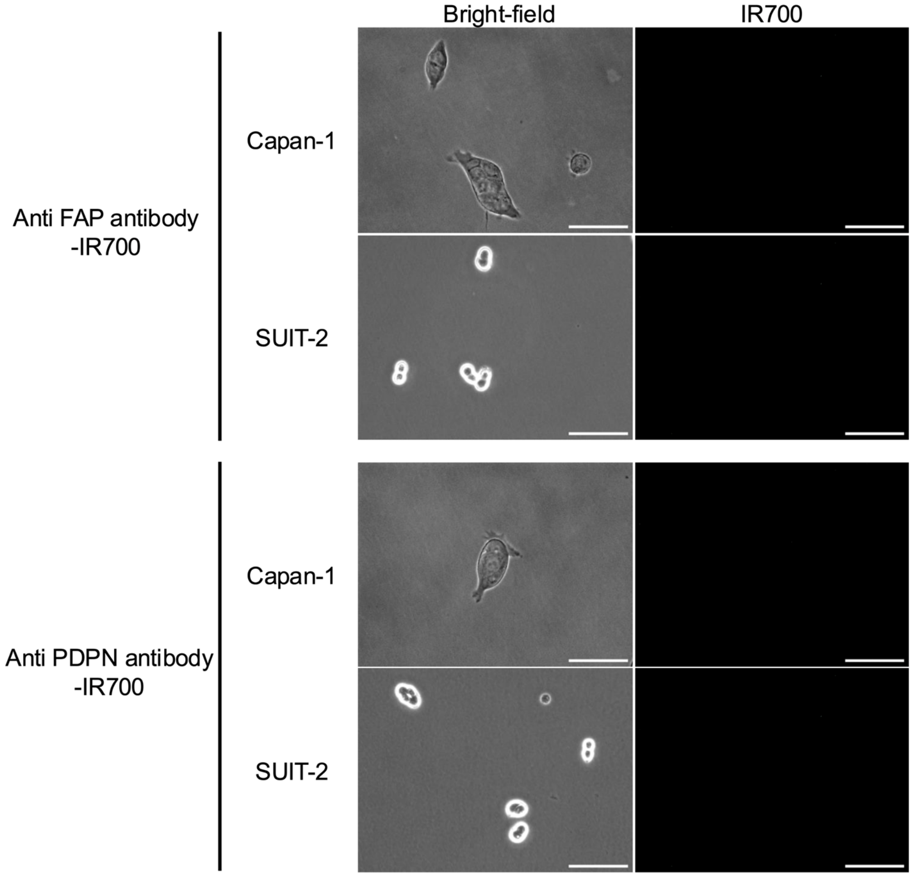

Figure S1. Fluorescence microscopy for confirmation of binding of anti-FAP (PDPN) antibody-IR700 conjugate to PDAC cells. In fluorescence microscopy, fluorescence due to binding of anti-human FAP (PDPN) antibody-IR700 conjugate to PDAC cells was not observed.

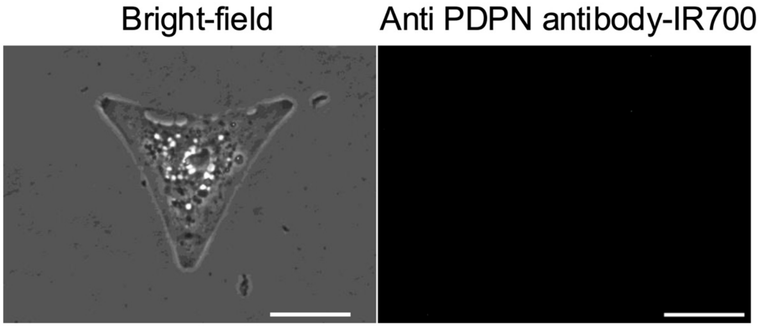

Figure S2. Fluorescence microscopy for confirmation of binding of anti-PDPN antibody-IR700 conjugate to hPSC-5. In fluorescence microscopy, fluorescence due to binding of anti-PDPN antibody-IR700 conjugate to hPSC-5 was not observed.

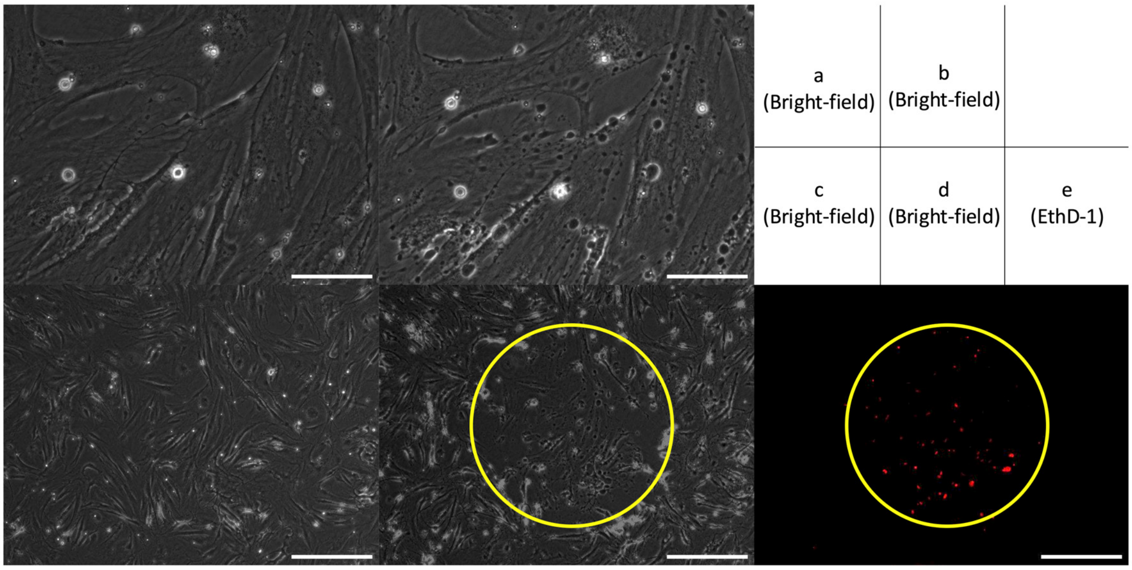

Figure S3. Microscopic observation after NIR-PIT *in vitro*. (**a, b**): Swelling was observed on the cell membrane surface immediately after NIR irradiation (a: before irradiation, b: immediately after irradiation; Scale bar: 100 μm, original magnification, 20X). (**c–e**): Cell death was observed only in the NIR irradiated area where is inside the yellow line. (c: Low power field image before irradiation, d: Three hours after irradiation in bright field, e: Three hours after irradiation in fluorescence field; Scale bar: 500 μm, original magnification, 4X)

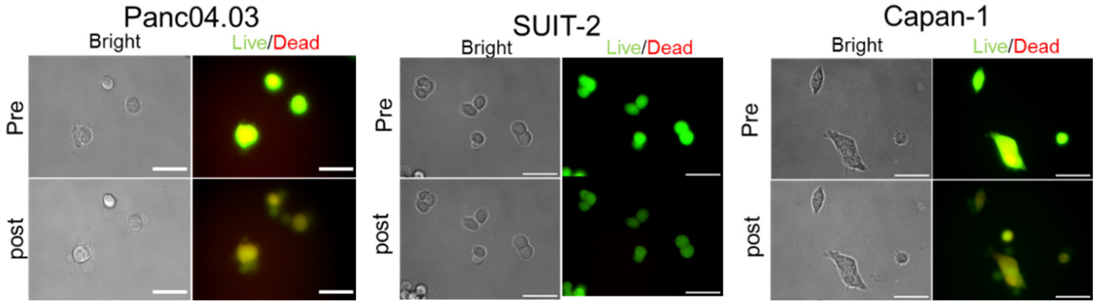

Figure S4. Microscopic observation of NIR-PIT for human pancreatic cancer cells (Panc04.03, SUIT-2, and Capan-1) using αFAP-IR700 in vitro. At 3 h after the end of irradiation, no cell swelling and no nuclear staining with EthD-1 were observed under a bright-field and fluorescence microscope, indicating no cell death. Scale bar: 50 μm.
